# Last mile research: a conceptual map

**DOI:** 10.1080/16549716.2021.1893026

**Published:** 2021-03-18

**Authors:** Colleen M. Davison, Susan A Bartels, Eva Purkey, Abigail H Neely, Elijah Bisung, Amanda Collier, Sherri Dutton, Heather M Aldersey, Kendall Hoyt, Chelsey L Kivland, Jennifer Carpenter, Elizabeth A Talbot, Lisa V Adams

**Affiliations:** a Department of Public Health Sciences, Queen’s University, Kingston, Ontario, Canada; b Department of Emergency Medicine, Queen’s University, Kingston, Ontario, Canada; c Department of Family Medicine, Queen’s University, Kingston, Ontario, Canada; d Department of Geography, Dartmouth College, Hanover, New Hampshire, USA; e School of Kinesiology and Health Studies, Queen’s University, Kingston, Ontario, Canada; f School of Rehabilitation Therapy, Queen’s University, Kingston, Ontario, Canada; g Geisel School of Medicine, Dartmouth College, Hanover, New Hampshire, USA; h Department of Anthropology, Dartmouth College, Hanover, New Hampshire, USA; i Center for Global Health Equity, Dartmouth College, Hanover, New Hampshire, USA

**Keywords:** Global health research, research methods, health equity, concept mapping, hard-to-reach populations, last mile

## Abstract

**Background**: The term ‘last mile’ has been used across disciplines to refer to populations who are farthest away, most difficult to reach, or last to benefit from a program or service. However, last mile research lacks a shared understanding around its conceptualization.

**Objectives**: This project used a concept mapping process to answer the questions: what is last mile research in global health and, how can it be used to make positive change for health equity in the last mile?

**Methods**: Between July and December 2019, a five-stage concept mapping exercise was undertaken using online concept mapping software and an in-person consensus meeting. The stages were: establishment of an expert group and focus prompt; idea generation; sorting and rating; initial analysis and final consensus meeting.

**Results**: A group of 15 health researchers with experience working with populations in last mile contexts and who were based at the Matariki Network institutions of Queen’s University, CAN and Dartmouth College, USA took part. The resulting concept map had 64 unique idea statements and the process resulted in a map with five clusters. These included: (1) Last mile populations; (2) Research methods and approaches; (3) Structural and systemic factors; (4) Health system factors, and (5) Broader environmental factors. Central to the map were the ideas of equity, human rights, health systems, and contextual sensitivity.

**Conclusion**: This is the first time ‘last mile research’ has been the focus of a formal concept mapping exercise. The resulting map showed consensus about *who* last mile populations are, *how* research should be undertaken in the last mile and *why* last mile health disparities exist. The map can be used to inform research training programs, however, repeating this process with researchers and members from different last mile populations would also add further insight.

## Background

Over the past two decades, the UN’s Millennium and Sustainable Development Goals (MDGs and SDGs) have focused on international intervention, research, and evaluation efforts and have produced much positive change in global health [1]. Member states signatory to the SDGs recognize the need to first reach those who are the farthest behind [2]. The ‘last mile’ is a phrase used to refer to people who are last to benefit from, furthest away from or hardest to reach with, health innovations and interventions. The United Nations Development Program (UNDP) defines that last mile populations as ‘not only the poorest of the poor, but also the people, places and small enterprise levels that are under-served and excluded, where development needs are greatest, and where resources are most scarce [1, p.8].’ Ensuring that the SDG commitments are translated into effective action for health equity for those in the last mile is a distinct challenge. While the United Nations will want to see that populations left behind during the MDG era do not remain in the same position by 2030, persistent health inequities remain a common challenge faced by health systems globally.

The COVID-19 pandemic has again highlighted the differential impacts of health risks globally and particular groups, including those living in poverty, have not benefitting equally from public health countermeasures [2,3]. Since the onset of the pandemic, there have been numerous concerns over backward slides with respect to health development and ongoing inequitable access to health opportunities for every sub-population [4–7]. It is largely recognized that any development targets will remain unreachable if we cannot address the health needs of *all* people, right down to the last mile. We require a more precise conceptualization of who might be considered last mile populations and what their needs and lived experience might be. We also have to have a better sense for how research best engages at or with the last mile. These concerns have been heightened in the era of COVID-19 but they exist as broad and present concerns that extend beyond the pandemic.

Unfortunately, in most contexts, disaggregated data for population sub-groups who might be facing health inequities, especially those in many last mile contexts are sparse. UNDP has recognized that with respect to the MDGs ‘few of the current indicators are able to shed light on the particular situations of migrants, refugees, older persons, persons with disabilities, minorities and indigenous peoples’ [8, p.1], for example. The phrase ‘No one is left behind’ is mentioned five times in the 2030 *Agenda for Sustainable Development*, but the challenge in meeting this ideal, and the implications of not meeting it, are significant and daunting [8].

### Defining the last mile

In a formal sense, the term ‘last mile’ comes originally from business and technology sectors where it represents the last groups and locations receiving a service or product [9,10]. The June 2018 UN Global Solutions Summit in New York City, for example, focused on technology deployment from the ‘lab to the last mile’ and the last mile was described as the final stages and populations in the diffusion of innovations [11]. Some global health researchers and practitioners have adopted the term ‘last mile’ to refer to those populations hardest to reach [12], or furthest away, from programs and services [13–15]. The UNDP has referred to specific population sub-groups that are more commonly in the last mile, including, for instance, people living in extreme poverty, refugee populations, the elderly, members of ethnic and religious minority groups, Indigenous Peoples or people living with disability, among others [1]. To add a layer of complexity, some population sub-groups may be considered within the ‘last mile’ in one context or with respect to a specific health or social issue, but not others. There may also be heterogeneity within these groups and not every member is equally positioned with respect to any particular ‘last mile’ [13,14].

Despite the growing usage of the term last mile, there is no consensus on its conceptualization [1]. In addition, those aiming to document last mile health inequities through data collection and research have not reached consensus about how best to do this or indeed what last mile research or last mile research methods are specifically [9,12].

Information gaps often exist with respect to last mile contexts because populations in the last mile can be difficult to reach geographically [16], may not commonly volunteer to be involved in research or evaluations [6], may be stigmatized [17], may have had negative experiences with research in the past [18,19], may not be easy to find [20,21], may be difficult to communicate with [22] or may not remain involved in research or evaluation projects over time [23]. Last mile environments may be areas of conflict and unrest, remoteness, or extreme poverty, making research and evaluation in these places challenging from logistical and resource perspectives [24,25]. Women and girls are majority members of many last mile populations because of sexism, exclusion, discrimination, and the systemic gender inequalities that leave them farthest behind [26]. Racial or ethnically marginalized populations are also over-represented among last mile populations [27,28].

To date, although valuable initiatives exist, including the UNDP’s ‘Getting to the Last Mile in Least Developed Countries’ [1], last mile research and evaluation remains fragmented [12] and has not yet benefited from advancements that could be gained through a shared understanding of how research can better engage with, and make positive change among last mile populations [12–15]. While being aware of population and contextual differences, a shared understanding of ‘last mile’ and ‘last mile research’ may facilitate networking and the sharing of methods, tools, and experiences more broadly amongst researchers working with and across populations in the last mile.

### Objective

We endeavored to undertake a concept mapping exercise for ‘last mile research’ with a group of health researchers who have experience doing research with people who could be considered as being in the ‘last mile’. Our main objective was to develop a conceptualization of ‘last mile research’ so we could inform further methodological and practical discussions about making positive change for health equity in the last mile.

## Methods and materials

### Concept mapping study design

Concept mapping is a way of identifying essential components of a concept and then assembling these in a way that can help in conceptual understanding. As a study design, it has been used in many areas including hand hygiene [29], intimate partner violence [30], gender and higher education [31], healthy aging [32], medical education [33], community-based housing [34], health care management [35] and understanding health care access barriers [36], among others. Traditionally, concept mapping is done over a series of steps or stages using tools such as paper, posters, post-it-notes and markers to engage groups in the process of cooperative conceptualization. While there is some methodological variation, the concept mapping process often includes five basic steps [37]. Step one is the establishment of a core research group, and the statement of a research question or opening prompt. The second step is to identify and invite people to participate in the mapping process (e.g. experts, representative stakeholders, or others). Participants react to the opening prompt or question by providing any number of unique statements or ideas in response. The third step is to remove duplicate statements or ideas and then have each participant ranks the statements in order of importance and works to group like statements into meaningful ideas or categories. Participants also provide potential labels for their categories. Finally, as a fifth step, a group meeting is held and responses (ranking and proposed categories) are discussed to develop a final concept map by consensus. Authors have noted that concept mapping is a participatory process that has the ability to initiate discussion, integrate knowledge across multiple disciplines, support consensus-building [37], interprofessional and group learning, and produce useful visual representations of ideas and their components [38,39]. More recently, online options have become available and these allow at least some of the stages to be done on a computer or handheld device. Including digital and even virtual aspects to a concept mapping process can enhance inclusion of people who are physically distant, it also allows for more easy quantification of some of the components, such as average ranks or proportion of co-sorting between any pair of ideas [40]. To date, this kind of concept mapping process has not been used to examine the concepts of hardest to reach, people left behind, last mile or last mile research. We believe that this kind of mapping and consensus-building exercise could help advance our conceptual understanding as well as provide a platform for informing further actions to address last mile health inequities.

Therefore, to meet our project objective, we undertook a concept mapping exercise using online concept mapping software and an in-person consensus meeting. The entire process occurred between July – December 2019. This research project was reviewed and given scientific and ethical clearance from the Queen’s University Health Sciences Research Ethics Board.

### Stage one: determining a focus prompt and convening a group

The first step in the concept mapping exercise was to establish a core research team (including authors CD and LVA as well as a software advisor from Group Wisdom). The project was part of a Matariki Network development initiative between Queen’s University in Kingston, Ontario, Canada and Dartmouth College, Hanover, New Hampshire, USA and only members of these institutions took part in this concept mapping exercise. The focus prompt for this project was: ‘*Last mile research in global health includes, or should include, factors and characteristics such as …’*. This project aimed to gather ideas from health researchers (‘experts’) who have experience working with last mile populations, in last mile contexts, or on health issues of last mile populations by any definition. Recruitment emails were sent out across health research networks at Queen’s and Dartmouth asking for volunteers. A formal quantitative, sample size calculation was not computed for this study as it was largely a qualitative approach. We followed guidance from Kane and Rosas [40] and selected a minimum of 15 participants who were knowledgeable experts or stakeholders on the topic. We also aimed for a sample with good variation in geographical area of research focus, research topics, discipline, and career stage. We recognized that this mapping exercise likely represented a first stage of what ideally would include a larger and more geographically diverse group of researchers and representatives from last mile populations themselves.

### Stage two: inviting experts into an online brainstorming activity

A license was obtained for group concept mapping software through Group Wisdom – Concept Systems Inc (https://groupwisdom.com/). A project space was established and basic demographic questions (i.e. university location, type of research undertaken, geographic area of focus, population of focus, disciplinary background, career stage) and focus prompt were uploaded. Researchers who were eligible, agreed to participate in the concept mapping, and consented to being part of the overall project were sent a hyperlink to connect to the Group Wisdom project webspace. These experts logged in and as a first step they engaged in brainstorming. They were provided with the following prompt: ‘*Last mile research in global health includes, or should include, factors and characteristics such as …’* and were asked to provide as many short written statements to complete the focus prompt as they could think of. This stage was open for two weeks and at the end of this time, a list of all statements, with duplications, was compiled.

### Stage 3: consolidating statements and inviting experts online to sort and rank

The list of brainstorming statements was reviewed and obvious duplications were deleted by members of the core research team. Once a consolidated list was ready, a second invitation was sent to the group asking the researchers to create their own categories or groups of ideas and to sort the individual statements into these categories. Any number of categories was allowable. The researchers were asked to label their own categories. Following the sorting, researchers were also asked to rank from their perspectives for each statement with regards to (a) importance and (b) current presence with 1 being none at all and 5 being very high.

### Stage 4: initial analysis – determining possible solutions

An initial analysis of the statements was undertaken by members of the core research team using data from the sorting exercise to draw a similarity matrix. This matrix is constructed by listing the statements across both the rows and columns in a table, and inputting values representing the co-sorting frequency for each pair of statements. Two statements that are commonly sorted together will have higher values in their crossing cell than two statements that are never or infrequently sorted together. The range of possible values in the similarity matrix cells is from 0 (no one sorted them together) to the maximum total number of participants (everyone sorted them together) [40].

The similarity matrix provides the dataset that unpins the creation of a two-dimensional array or point map. Non-metric, multidimensional scaling (MDS) is used to create a two-dimensional representation of the similarity matrix data. Non-metric MDS is a technique that converts the relative similarity (or dissimilarity) of two items into a relative distance between these items. Thus, each statement has a specific ‘distance’ from all other statements, and each statement has a unique relationship to all the other statements in the full group. An MDS introduction [41] and MDS algorithm details [42] are provided elsewhere. Within the Group Wisdom software, the MDS algorithm helps to calculate two-dimensional coordinates for each statement and these are then plotted in an array or point map.

**Figure 1.**
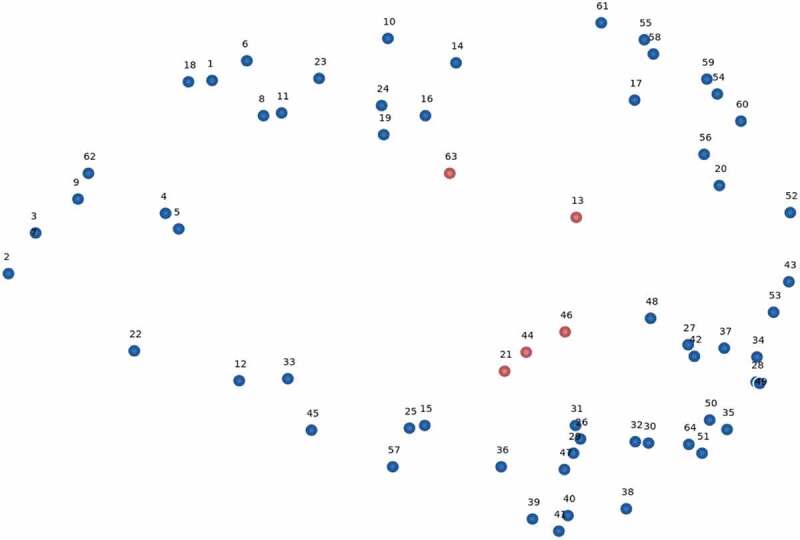
Conceptual point map of 64 statements in relative location based on number of times statements were co-sorted by 15 independent global health researchers.*

**Figure 2.**
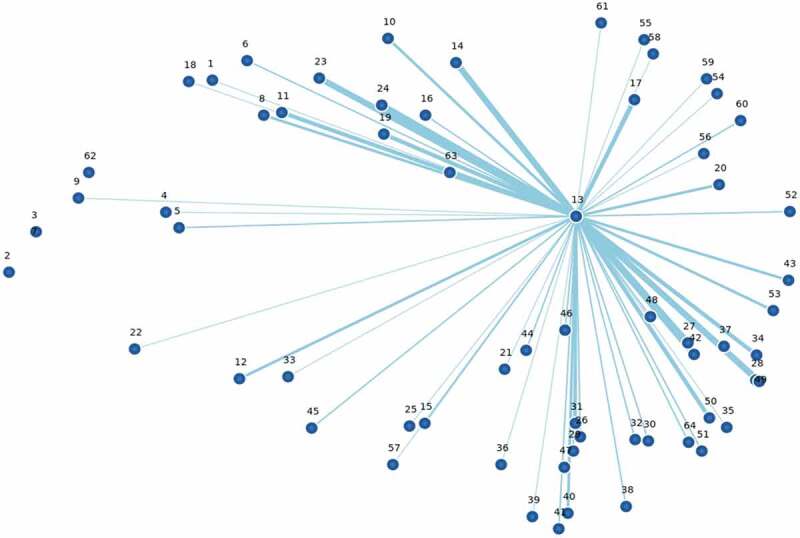
Point map of the project statements showing the central location of the statement: ‘*13. Understanding the barriers to equitable health from the perspective of people in the last mile*’.*

Kane and Rosas (2018) describe point maps as constellations of ideas that are captured in space [40]. Point maps (as seen in Figures 1, 2 and 4) are useful since statements are depicted in relative location to one another. The north-south-east-west orientation of the point map is not fixed. The map is especially useful to show the central or peripheral nature of specific statements and potential clusters of points.

*Numbers correspond to the numbered text statements provided in Table 2. Centrally located points noted by the group are highlighted in red.

*Lines indicate number of times statement #13 was sorted in a similar conceptual group with other statements. Thinnest line indicates 1–2 times sorted together. Thickest lines indicate 13–15 times sorted together.

The next step in the concept mapping analysis was to categorize statements as depicted in the two-dimension point map into useful or meaningful groups or clusters. Within the Group Wisdom software, agglomerative hierarchical cluster analysis (HCA) is used [43,44]. Using this approach, each statement begins in its own cluster and then is merged with the statistically closest two statements, then the next two and so on. Ward’s algorithm[45] provides a decision-rule to help divide the points on the map into statistically non-overlapping clusters. It is possible to determine the number of clusters you would like to test apriori. We chose to examine cluster solutions with 3–11 clusters so that we would have a range of granularity in the possible idea groupings.

### Stage 5: in-person meeting to complete analysis and achieve consensus

The team of researchers met in person over two-days in December 2019 to discuss the cluster solutions that were created, interpret the meanings of the clusters, determine cluster labels and decide upon the most clear and relevant cluster solution that would represent the group’s final concept map.

## Results

### Overview

Nineteen researchers came forward following the recruitment at each University. All were eligible and invited to the online space where the concept mapping was to occur. In the end, 15 global health researchers completed all of the online concept mapping exercises and attended the consensus meeting. Table 1 outlines the demographic characteristics of these researchers.

**Table 1.** Characteristics of the global health researchers who undertook the concept mapping process*

Characteristics** [n(%)]			
Career Stage Early Middle Later	3 (20.0) 9 (60.0) 3 (20.0)	Population of Research Focus Ethnic minority Indigenous persons People living in extreme poverty Individuals with stigmatizing disease (e.g. HIV, Ebola) Refugees and Internally Displaced Remote Populations Persons Living with Disability Other	2 (13.3) 1 (6.7) 5 (33.0) 4 (26.7) 4 (26.7) 2 (13.3) 1 (6.7) 1 (6.7)
Type of Research Quantitative Qualitative Community-Based, Participatory Mixed-Methods	5 (33.3) 4 (26.7) 3 (20.0) 5 (33.3)	Research Locations of Focus Australia and Region Central and Northern Asia Europe North Africa and the Middle East Populations in the USA Populations in Canada Central and South America Southern Asia South East Asia Sub-Saharan Africa Global or not a specific continent No geographic focus	0 (0.0) 1 (6.7) 0 (0.0) 1 (6.7) 0 (0.0) 4 (26.7) 0 (0.0) 0 (0.0) 3 (20.0) 3 (20.0) 7 (46.7) 4 (26.7) 1 (6.7)
Disciplinary Background Anthropology Clinical Medicine Economics Environmental Studies Epidemiology Ethics Gender Studies Geography Political Studies Public & Population Health Research Methodology Sociology	2 (13.3) 5 (33.3) 1 (6.7) 2 (13.3) 7 (46.7) 2 (13.3) 4 (26.7) 2 (13.3) 2 (13.3) 7 (46.7) 7 (46.7) 2 (13.3)	Current University Location Queen’s University, CANADA Dartmouth College, USA	9 (60.0) 6 (40.0)

*Each participant self-identifies as a researcher who engages with people who might be considered ‘last mile populations’ in some conceptualization.

**Participants chose any responses that applied, thus columns do not always add to n = 15.

After statements with duplicate meaning were removed, the process resulted in 64 unique statements in response to the prompting question: ‘*Last mile research in global health includes, or should include, factors and characteristics such as …’* Table 2 is the list of these statements.

**Table 2.** The list of 64 unique statements provided by 15 global health researchers when prompted ‘*Last mile research in global health includes, or should include, factors and characteristics such as…’*

1. economic barriers at personal, social and system levels. 2. the effect of climate change on the area and people. 3. conflicts and how they affect the population. 4. consideration of social determinants of health. 5. examining the determinants of health and how to improve these factors. 6. consider structural determinants of health. 7. displacement and its effects. 8. looking upstream for factors that have led to the group being ‘last mile.’ 9. how geographic isolation affects the population. 10. social marginalization. 11. overcoming obstacles that have limited inclusion of last mile groups traditionally. 12. defining and measuring obstacles to desired health outcomes. 13. understanding the barriers to equitable health from the perspective of people in the last mile. 14. understanding stigmatization in the context. 15. access to research, services and information gathering to inform data & services. 16. understanding the community and whether one group is making another vulnerable. 17. the diversity of disability & engagement needs. 18. the influence of governmental structures and policies at the local level. 19. attention to reasons why the population is considered a ‘last mile’ population. 20. a focus on populations who have not always benefited from research or interventions before. 21. being culturally and contextually sensitive. 22. the centralization of public workers and services. 23. understanding the systemic biases and processes that contribute to individuals or groups being ‘last mile.’ 24. examining the systems of power that have led to marginalization. 25. mapping of transportation options, cost, frequency, and hazards. 26. generated, conducted and interpreted by those with full understanding of cultural, social, fiscal context. 27. empowerment of participants and communities. 28. community engagement throughout all stages of research 29. universal design for engagement principles. 30. participatory methods that can be conducted remotely. 31. equity centered research questions, methods, and sampling strategies. 32. methods that might have to be adapted or designed specifically.	33. addresses issues that encompass the entirety of patient cascade (prevention, treatment, health maintenance) 34. priorities that have been identified by last mile populations themselves. 35. reflexive thinking and practice throughout the research process. 36. multi-stakeholder buy-in from the beginning to extent possible. 37. attention to power dynamics in research. 38. a way to return research results to the affected population. 39. a focus on how research can be translated into action or improvements for last mile populations. 40. translation of research to build capacity and knowledge. 41. being thoughtful about integrative and end-of-grant knowledge translation. 42. community activism. 43. advocacy so that people in the last mile are not ‘left behind’ in development or health gains. 44. intentionally promoting health equity. 45. equity or fairness. 46. a focus on human rights. 47. being thoughtful around all ethical considerations. 48. social justice as an underlying value. 49. recognizing and valuing community members as experts in their own lives. 50. participatory approaches to meaningfully reach individuals and groups often overlooked or left out of traditional research. 51. telling stories and showing data that are often neglected. 52. finding and advocating for those without a political or social voice. 53. raising voices of the unheard. 54. children, women, the elderly and others in specific life stages relevant to the research. 55. people with disabilities, including invisible disabilities. 56. looking at specific hard to reach groups due to equity-related concerns. 57. involving local pharmacies, healers, midwives, birth-attendants. 58. indigenous people and any minority group in the context–religious, sexual, migrant, refugees etc. 59. most vulnerable populations that current efforts have not yet reached. 60. those who have traditionally been left out of ‘mainstream’ research. 61. populations who face multiple vulnerabilities that marginalize them further. 62. the security situation for vulnerable groups. 63. identifying the circumstances that have positioned people on the margins of healthcare systems. 64. using methods that privilege the voices of minority populations.

A relative distance point map was created (Figure 1).

We have highlighted five statement numbers in red as these are located centrally to the map.

#13 – understanding the barriers to equitable health from the perspective of people in the last mile.

#21 – being culturally and contextually sensitive.

#44 – intentionally promoting health equity.

#46 – a focus on human rights.

#63 – identifying the circumstances that have positioned people on the margins of healthcare systems

Statement #13 for example: ‘Understanding the barriers to equitable health from the perspective of people in the last mile’ was co-sorted with almost all of the other items across the map at least once. Lines in Figure 2 show frequency of co-sorting with the thinnest lines representing statement #13 being co-sorted 1–2 times with other statements, and the thickest lines represent it being co-sorted with other statements 13–15 times across the 15 researchers.

**Figure 3.**
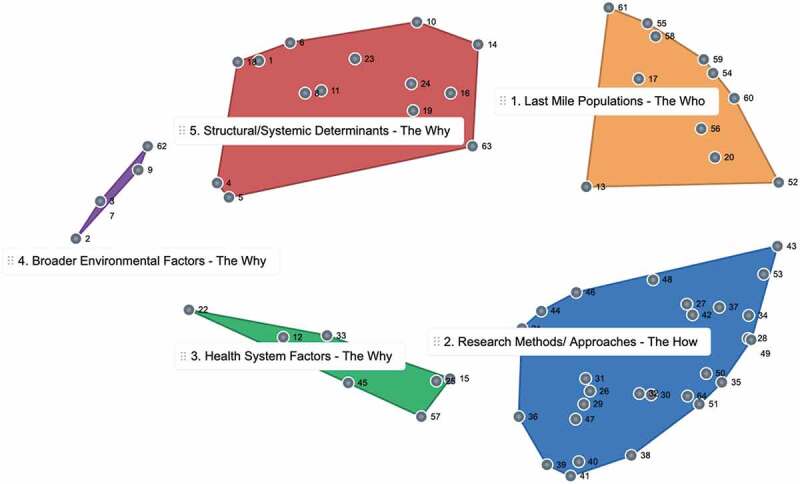
Final five cluster concept map for ‘last mile research’ determined through discussion and consensus with global health researchers

The group reviewed and discussed the point map and the potential 3–11 cluster concept map solutions. In the consensus meeting, the group considered the individual sorting results as well as qualitatively assessed the multiple potential solutions and determined that the five cluster solution was the most representative of the conceptual ideas of the group. A map with fewer clusters did not have sufficient granularity of ideas; having more than five clusters meant that broad areas were too finely divided. The participants also reached consensus about the labels for each of the identified five clusters (Figure 3, Table 3).

**Table 3.** Statements sorted into the five cluster areas

**Cluster 1: Last Mile Populations – The Who** 13. understanding the barriers to equitable health from the perspective of people in the last mile. 17. the diversity of disability & engagement needs. 20. a focus on populations who have not always benefited from research or interventions before. 52. finding and advocating for those without a political or social voice. 54. children, women, the elderly and others in specific life stages relevant to the research. 55. people with disabilities, including invisible disabilities. 56. looking at specific hard to reach groups due to equity-related concerns. 58. indigenous people and any minority group in the context–religious, sexual, migrant, refugees, and others. 59. most vulnerable populations that current efforts have not yet reached. 60. those who have traditionally been left out of ‘mainstream’ research. 61. populations who face multiple vulnerabilities that marginalize them further. **Cluster 2: Research Methods/Approaches – The How** 21. being culturally and contextually sensitive. 26. generated, conducted and interpreted by those with full understanding of cultural, social, fiscal context. 27. empowerment of participants and communities. 28. community engagement throughout research: conceptualization, design, implementation, dissemination. 29. universal design for engagement principles. 30. participatory methods that can be conducted remotely. 31. equity centered research questions, methods, and sampling strategies. 32. methods that might have to be adapted or designed specifically. 34. priorities that have been identified by last mile populations themselves. 35. reflexive thinking and practice throughout the research process. 36. multi-stakeholder buy-in from the beginning to extent possible. 37. attention to power dynamics in research. 38. a way to return research results to the affected population. 39. a focus on how research can be translated to action or improvements for last mile populations. 40. translation of research to build capacity and knowledge. 41. being thoughtful about integrative and end-of-grant knowledge translation. 42. community activism. 43. advocacy so that people in the last mile are not ‘left behind’ in development or health gains. 44. intentionally promoting health equity. 46. a focus on human rights. 47. being thoughtful around all ethical considerations. 48. social justice as an underlying value. 49. recognizing and valuing community members as experts in their own lives. 50. participatory approaches to meaningfully reach individuals and groups often overlooked or left out of traditional research. 51. telling stories and showing data that are often neglected. 53. raising voices of the unheard. 64. using methods that privilege the voices of minority populations.	**Cluster 3: Health System – The Why** **Factors – The Why** 12. defining and measuring obstacles to desired health outcomes. 15. access to research, services and information gathering to inform data & services. 22. the centralization of public workers and services. 25. mapping of transportation options, cost, frequency, and hazards. 33. addresses issues that encompass the entirety of the patient cascade from prevention to symptoms to restored health. 45. equity or fairness. 57. involving local pharmacies, healers, midwifes, birth-attendants. at personal, social and system levels. 6. consider structural determinants of health. 8. looking upstream for factors that have led to the group being ‘last mile.’ 10. social marginalization. 11. overcoming obstacles that have limited inclusion of last mile groups traditionally. 14. understanding stigmatization in the context. 16. understanding the community and whether one group is making another vulnerable or under-acknowledged. 18. the influence of governmental structures and policies at the local level. **Cluster 4: Broader Environmental Factors – The Why** 2. the effect of climate change on the area and people. 3. conflicts and how they affect the population. 7. displacement and its effects. 9. how geographic isolation affects the population. 62. the security situation for vulnerable groups. **Cluster 5: Structural/Systemic Determinants – The Why** 1. economic barriers 19. attention to reasons why the population is considered a ‘last mile’ population. 23. understanding the systemic biases and processes that contribute to individuals or groups being ‘last mile.’ 24. examining the systems of power that have led to marginalization. 4. consideration of social determinants of health. 5. examining the determinants of health and how to improve these factors. 63. identifying the circumstances that have positioned people on the margins of healthcare systems.

**Cluster 1**: Last Mile Populations – *The Who*.

**Cluster 2**: Research Methods and Approaches – *The How*.

**Cluster 3**: Health System Factors – *The Why*.

**Cluster 4**: Broader Environmental Factors – *The Why*.

**Cluster 5**: Structural/Systemic Factors – *The Why*.

*The final concept map* contains five clusters of varying sizes and was decided by consensus in the in-person meeting that this was the best representation of the groups’ ideas overall. There are three broad themes: The Who, The What, and the Why. The ‘why’ theme has three subthemes as described below.

## The who

### Cluster 1: last mile populations

The last mile populations cluster within Figure 3 contains 11 statements that refer to the ‘*who*’ with respect to last mile research. These statements emphasize the *people or populations* in the last mile such as people with disabilities or those who live in remote or conflict-affected settings. This cluster exists between the research methods area of the map, and the structural/socia determinants area. Statements that are at the edges of a specific cluster provide bridging ideas with the other clusters that they are oriented towards. For example, at the south and south-east end of this cluster are the statements:

13. understanding the barriers to equitable health from the perspective of people in the last mile.

52. finding and advocating for those without a political or social voice.

Both of these statements are oriented towards the research methods part of the map and highlight the need to find and learn directly from the perspectives of members of last mile populations directly and the need to engage in advocacy with these groups.

## The how

### Cluster 2: research methods and approaches

The research methods and approaches cluster contain 26 statements that speak to the *how* of last mile research. These are specific ideas about research methods and approaches for use with last mile populations. This is the largest cluster in the map. This cluster sits between the last mile populations cluster, and the cluster that includes statements about health system factors. Again, peripheral statements can help provide bridges between idea-areas on the map. The western edge of the research methods cluster includes the statement:

36. multi-stakeholder buy-in from the beginning to the extent possible.

As this statement is oriented towards the health services cluster, it highlights the need to meaningfully engage stakeholders, including those in the health system, in last mile research related to global health issues.

The three remaining clusters speak to why last mile populations exist on the margins, why they experience health inequities or why specific approaches might be needed in last mile research.

## The why

### Cluster 3: health system factors

The next cluster of the map is the health system factors cluster, shown in Figure 3, which contains seven unique statements. The focus prompt asked specifically about last mile research in global health, and participants added statements specific to health systems that were often co-sorted together and not co-sorted with other ideas. Examples of health cluster statements included:

15. access to research, services and information gathering to inform data & services.

25. mapping of transportation options, cost, frequency, and hazards.

33. addresses issues that encompass the entirety of patient cascade (prevention, treatment, health maintenance)

57. involving local pharmacies, healers, midwives, birth-attendants.

Although statements 15 and 25 do not specifically mention ‘health systems’ the participants commonly co-sorted these statements into this cluster. In discussion, participants stated that they presumed that the understanding for #15 was ‘health services’ and for #25 that transportation options must refer to transportation when attempting to access health services.

### Cluster 4: broader environmental factors

The smallest cluster on the map is the environmental cluster, which contained five statements in the farthest western region of the concept map. These statements are broad environmental determinants, or reasons *why* last mile populations may exist, be difficult to reach or require specific approaches from a research perspective:

2. the effect of climate change on the area and people.

3. conflicts and how they affect the population.

7. displacement and its effects.

9. how geographic isolation affects the population.

62. the security situation for vulnerable groups.

### Cluster 5: structural/systemic determinants

The structural/systemic determinants cluster at the north of the concept map is the second largest section of the map and includes 14 statements that speak to the structural and systemic determinants of health or of being among last mile populations. These include for example:

1. economic barriers at personal, social and system levels.

8. looking upstream for factors that have led to the group being ‘last mile.’

11. overcoming obstacles that have limited inclusion of last mile groups traditionally.

16. understanding the community and whether one group is making another vulnerable.

23. understanding the systemic biases and processes that contribute to individuals or groups being ‘last mile.’

### Rating results

**Figure 4.**
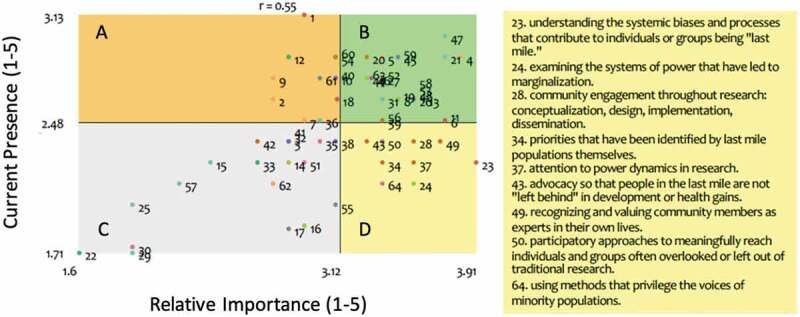
Identifying next steps- average level of importance and current presence ratings for 64 ‘last mile research’ statements as determined by 15 global health researchers.*

The researchers rated each statement for its importance and current presence on a 1 (not at all) to 5 scale (very high). Average ratings were plotted on an x/y grid, with statements then categorized into four quadrants (high-high, high-low, low-high, low-low) (Figure 4).

*The graph indicates average rating among the 15 participants (1 not at all – 5 very high) for importance and current presence of each of the 64 statements. The correlation coefficient **r** measures the strength and direction of any linear relationship between the two variables. The yellow quadrant (D) indicates those statements that have high relative importance but currently low presence. These statements are listed to the right of the diagram and represent potential next steps.

The statements that are in the D quadrant, which indicates that on average researchers rated them of high importance, but low presence, are:

23. understanding the systemic biases and processes that contribute to individuals or groups being ‘last mile.’

24. examining the systems of power that have led to marginalization.

28. community engagement throughout all stages of research

34. priorities that have been identified by last mile populations themselves.

37. attention to power dynamics in research.

43. advocacy so that people in the last mile are not ‘left behind’ in development or health gains.

49. recognizing and valuing community members as experts in their own lives.

50. participatory approaches to meaningfully reach individuals and groups often overlooked or left out of traditional research.

64. using methods that privilege the voices of minority populations.

This is the quadrant that indicates areas for potential focus and future action from the perspective of the health researcher participants.

## Discussion

A group of 15 health researchers based at two Matariki Network Universities completed a five-stage concept mapping exercise between July and December 2019. The resulting concept map had 64 unique idea statements grouped in five clusters. This is the first time ‘last mile research’ has been the focus of a formal concept mapping exercise and the process highlighted a number of important themes. First, central to the concept of last mile research are the ideas of health equity, human rights, being culturally and contextually sensitive and understanding inherent barriers in health care systems. These ideas were positioned in the middle of the map, indicating that they were often co-sorted with other ideas across all sections of the map. Health equity is created when individuals have the fair opportunity to reach their fullest health potential [46]. Supporting health equity is working to ensure fairness in access to health outcomes and opportunities across all people, including those in the last mile. Human rights are defined through the Universal Declaration of Human Rights [47] as well as subsequent documents describing rights of specific groups such as Indigenous Peoples [48], children [49], or people living with disabilities [50]. For decades human rights and health equity have been central to global health research conceptualizations and endeavors more broadly and have been used as important concepts and frameworks on which to make advancements. For the researchers in this study who are engaging with populations and issues in the last mile, equity and human rights remain pivotal and this finding aligns well with equity-centered global health research described across the literature [16,26,46,51].

The researchers in this study also placed the culture, context, views, and experiences of people in the last mile as central to the concept of last mile research. This came out in the statements such as: *understanding the barriers to equitable health from the perspective of people in the last mile; priorities that have been identified by last mile populations themselves; and overcoming obstacles that have limited inclusion of last mile groups traditionally*. Last mile populations are thus appropriately positioned as the rightful experts in their own lives and the most important stakeholder in any last mile research. The research methods and approaches cluster emphasizes participatory approaches, as well as knowledge translation and partnership. At the margins between the research methods cluster and the last mile populations cluster is the idea of ‘advocacy so that people in the last mile are not left behind in development or health gains’. Advocacy is about building support for a specific idea or cause. Advocating for change in the last mile may involve the use of specific types of research data to persuade decision-makers, but it may also use integrated knowledge translation which positions decision-makers centrally in the research, alongside researchers and members of last mile populations, from the outset [52]. While advocacy is critical for creating change particularly when the status quo may benefit those currently in power to make change, effective advocacy is often not central to the curriculum in health-related research or professional programs and we believe requires a greater focus on research training programs [53]. Capacity development with last mile populations is essential to ensure that the right skills exist from within to address current and future barriers to health equity [1].

While advocacy was one idea that bridged the ‘who’ and ‘how’ clusters of the map, the idea of ‘identifying the circumstances that have positioned people on the margins of healthcare systems (#63)’ bridged three areas of the map: structural and systemic factors; last mile populations; and health care factors. These bridging ideas are key action areas for addressing last mile health inequities and in our thinking about ways to enhance training for last mile researchers. Strengthening national health systems has been a key approach used over the past 30 years to ensure health for all [54–56]. These efforts include initiatives for ensuring access to essential medicines, establishing health financing mechanisms to ensure universal access, the development of functioning medical records systems, the training of a strong and diverse health workforce and the provision of safe and accessible services [49]. Although researchers have historically been involved in these endeavors, it could be valuable for them to further increase focus on documenting and evaluating health system strengthening with the goal of health for all, particularly as we strive to reach populations in the last mile. Also inherent in idea #63 is a focus on underlying social and societal determinants of health. Populations are positioned at the margins of health care systems because of poverty, stigmatizing situations, disability, the impacts of colonization, and more. Last mile researchers must be sensitive to these ‘causes of the causes’ [51] in order to be most effective.

The rating exercise highlighted specific areas that are of high importance but have low presence in current research practice. Among the ideas highlighted by the group were community engagement, attention to power dynamics in research, participatory approaches and using methods that privilege the voices of minority populations. This may require entirely novel ways of collecting data or researchers adopting methods that are new to them. Last mile research prioritizes the voice and dignity of people who live in last mile situations and spaces. Researchers must engage closely with these communities using participatory approaches. Finding ways to do this in some forms of research, such as large population-based studies, may be a challenge, but there is precedence that can be instructive [57–60].

Most peripheral to the left side of the map is a small cluster containing broader environmental factors such as climate change, conflict, or displacement as they relate to last mile research in global health. This cluster was positioned relatively closely to the structural and systemic factors cluster indicating the similarities across these two clusters conceptually. Although there were variations between participants in the sorting of the broader environmental items, it was not common for them to be co-sorted with research methods and approaches ideas or with last mile populations ideas. This is why the broader environmental factors are furthest away from both of these clusters in the map. The distance between the broader environmental ‘why’ cluster and the ‘who’ and ‘how’ clusters seems to indicate the need, at least among the participants in this study, for increased awareness about last mile populations affected by broader environmental factors as well as further development or alignment of research methodological innovations focused on these issues and populations.

Our preliminary discussions suggested that we did not necessarily share a common definition of last mile populations and research at the outset. Through the process of concept mapping and consensus building, we were able to develop a concept map for ‘last mile research’ that can be used in further discussions and research methodological developments. For instance, the ideas from this concept map help identify why last mile populations are positioned at societal margins and how researchers could better design studies to acknowledge and include populations which may otherwise be missed. We believe ongoing discussions about last mile research are necessary in order to reach, engage with and support populations and health in the last mile.

### Strengths and limitations

This concept mapping exercise using supportive software had a number of strengths. The brainstorming, sorting, and rating stages were able to be done remotely and this allowed participation from any number of participants and across geographic areas. The initial use of the online platform helped in the creation of the concept map draft that was later discussed and interpreted by the group in person. The map showed clusters of ideas, and also the degree of centrality of some statements. The software helped to easily identify the relationships between statements so that a meaningful point map could be created. Maps with different numbers of clusters were possible and this was helpful as there were different levels of granularity of ideas that could make up the whole concept, the group was able to deliberate around this and select the best cluster solution.

In addition to strengths, the study did have some limitations. Our group was only partially diverse with respect to different characteristics that could be relevant to the topic (age, sex, geographic area of focus, nationality, university location etc). This is a significant limitation of the present work which should be seen as a first step. We believe that an important next step would be to conduct a similar mapping process with a more diverse set of global health researchers, as well as members of last mile populations themselves. Comparisons of maps created by different groups could be very insightful and help propel further conceptual understanding. With respect to the online software, the software license fee may make the use of the software inaccessible for some teams with limited resources, technological literacy and internet access may also be barriers. In addition, our team felt the in person consensus meeting was critical to producing a strong final concept map by consensus. While a virtual consensus meeting may be possible, we were not certain that it would have resulted in the same product as it was an interactive process aided by in person discussion and familiarizing ourselves with the other team members. Again teams with financial, geographic, or other constraints may not be able to conduct the in-person meeting.

## Conclusion

Our conceptual map of ‘last mile research’ emphasized the ‘who’ of last mile populations, the ‘how’ of specific research methods and approaches, and the ‘why’ of structural and systemic determinants, health system factors, and broader environmental factors. A group of 15 health researchers who are engaged in last mile research and who are located at two North American universities created a map that we believe could be used as a starting point for further discussions. Ideally, this concept would be further developed and clarified by last mile populations themselves as well as by a much wider diversity of researchers.

If we have determined some ideas about the who, how, and why of last mile research, it is certain that the answer to the ‘when’ is *now*. We are facing not only the COVID-19 pandemic, but more frequent and devastating natural and humanitarian disasters as well as widening inequities related to gender, race, religion, and other socioeconomic characteristics. Populations in the last mile face a unique set of health barriers and researchers engaging with them must contend with additional complexities inherent in last mile settings and spaces. Now more than ever, we must address the complexities of collecting data, engaging with populations and translating research into solutions at the last mile. We believe this last mile research conceptual map can help inform further methodological scholarship and practical developments towards these very important goals.

## Paper context

The term the ‘last mile’ is often used in public and global health research discussions. This paper outlines a concept mapping process undertaken to articulate ‘last mile research’ among global health researchers at one American and one Canadian university. The concept map denotes the who, the how and the why of last mile research in global health and represents the beginning of a longer conversation that is required among global health researchers more broadly.

## References

[1] Pedrajas M, Choritz S. Getting to the last mile in least developed countries. New York: UNDP; 2016. Available from: https://www.undp.org/content/dam/undp/library/SDGs/English/getting-to-the-last-mile-oct-2016.pdf

[2] International Committee of the Red Cross (ICRC). COVID-19: how IHL provides crucial safeguards during pandemics. Geneva: ICRC; 2020 [cited 2020 Jun 6]. Available from: https://www.icrc.org/en/document/covid-19-how-ihl-provides-crucial-safeguards-during-pandemics

[3] International Rescue Committee (IRC). COVID-19 in humanitarian crises: a double emergency. New York: IRC; 2020 [cited 2020 Jun 7]. Available from: https://www.rescue.org/report/covid-19-humanitarian-crises-double-emergency

[4] Corburn J, Vlahov D, Mberu B, et al. Slum health: arresting COVID-19 and improving well-being in urban informal settlements. J Urban Health. 2020;97:348–14. 32333243 10.1007/s11524-020-00438-6 PMC7182092

[5] Fuhrman S, Kalyanpur A, Friedman S. Gendered implications of the COVID-19 pandemic for policies and programmes in humanitarian settings. BMJ Glob Health. 2020;5:1–5. 10.1136/bmjgh-2020-002624 PMC7234868 32414748

[6] McKee M, Stuckler D. COVID-19 in humanitarian settings and lessons learned from past pandemics. Nat Med. 2020 May;26:647–648. 32269357 10.1038/s41591-020-0851-2

[7] CARE. Gender Implications of COVID-19 outbreaks in development and humanitarian settings. 2020 Mar 16 [cited 2020 Jun 6]. Available from: https://www.care-international.org/files/files/Gendered_Implications_of_COVID-19_Full_Paper.pdf

[8] United Nations Department of Economic and Social Affairs [Internet]. Sustainable development goals report. New York: United Nations; 2016 [cited 2020 Sept 3]. Available from: https://unstats.un.org/sdgs/report/2016/leaving-no-one-behind

[9] Olsson J, Hellstrom D, Palsson H. Framework of last mile logistics research: a systematic review of the literature. Sustainability. 2019;11:7131.

[10] Chao TE, Lo NC, Mody GN, et al. Strategies for last mile implementation of global health technologies. Lancet Glob Health. 2014;2:e497–e498. 25304406 10.1016/S2214-109X(14)70253-0

[11] Watkins A. Global solutions summit 2018. New York: United Nations; 2018 [cited 2020 Jun 15]. Available from: http://www.globalsolutionssummit.com/uploads/3/1/5/5/31554571/gss_2018_background_note_–_final.pdf

[12] Bonevski B, Randell M, Paul C, et al. Reaching the hard-to-reach: a systematic review of strategies for improving health and medical research with socially disadvantaged groups. BMC Med Res Methodol. 2014;14:42. http://www.biomedcentral.com/1471-2288/14/42 24669751 10.1186/1471-2288-14-42 PMC3974746

[13] Taylor W. The last mile in community health: reaching the hardest to serve. Health Affairs, Health Affairs Blog. 2015 [cited 2020 Aug 31]. Available from: https://www.healthaffairs.org/do/10.1377/hblog20150811.049892/full/

[14] Bill and Melinda Gates Foundation. Speech by Bill Gates at the reaching the last mile global health forum. Abu Dhabi, United Arab Emirates; 2017 Nov 15 [cited 2020 Jun 6]. Available from: https://www.gatesfoundation.org/Media-Center/Speeches/2017/11/Bill-Gates-Reaching-the-Last-Mile-Global-Health-Forum

[15] Last Mile Health. Boston: Last Mile Health; 2020 [cited 2020 Jun 6]. Available from: https://lastmilehealth.org

[16] AMFAR Aids Research. The shifting global health landscape: implications for HIV/AIDS and vulnerable populations. Issues Brief. New York: AMFAR The Foundation for Aids Research; 2010 Jul [cited 2018 Jun 20]. Available from: http://www.amfar.org/uploadedFiles/_amfarorg/Around_the_World/Vulnerable-Populations.pdf

[17] Hatzenbuehler ML, Phelan JC, Link BG. Stigma as a fundamental cause of population health inequalities. Am J Public Health. 2013;103:813–821. 23488505 10.2105/AJPH.2012.301069 PMC3682466

[18] WIison D, Neille S. Culturally safe research with vulnerable populations. Contemp Nurse. 2009;33:69–79. 19715497 10.5172/conu.33.1.69

[19] Reverby SM. Ethical failures and history lessons: the U.S. Public Health Service research studies in Tuskegee and Guatemala. Public Health Rev. 2012;34:13. 10.1111/bioe.12784 32608027

[20] Wahoush EO. Reaching a hard-to-reach population such as asylum seekers and resettled refuges in Canada. Bull World Health Organ. 2009;87:568–568. 19705000 10.2471/BLT.08.061085 PMC2733262

[21] Ellard-Gray A, Jeffrey NK, Choubak M, et al. Finding the hidden participant: solutions for recruiting hidden, hard-to-reach, and vulnerable populations. Int J Qual Methods. 2015;14:1–10.

[22] Stenson AL, Kapungu CT, Geller SE, et al. Navigating the challenges of global reproductive health research. J Women’s Health. 2010;19:2101–2107. 10.1089/jwh.2010.2065 PMC3004132 20849297

[23] Geraghty AW, Torres LD, Leykin Y, et al. Understanding attrition from international internet health interventions: a step towards global eHealth. Health Promot Int. 2013;28:442–452. 22786673 10.1093/heapro/das029 PMC3738071

[24] Byrne A, Hodge A, Jimenez-Soto E, et al. What works? Strategies to increase reproductive, maternal and child health in difficult to access mountainous locations: a systematic literature review. PLoS ONE. 2014;9:e87683. 24498353 10.1371/journal.pone.0087683 PMC3912062

[25] Moss SM, Uluğ ÖM, Acar YG. Doing research in conflict contexts: practical and ethical challenges for researchers when conducting fieldwork. Peace Conflict: J Peace Psychol. 2019;25:86–99.

[26] Blum Centre for Developing Economies. Fighting for the last mile of women’s rights: the Head of UN Women and UC Berkeley scholars look towards 2030. Berkeley: Blum Centre for Developing Economies; 2020 [2014 Dec 11] [cited 2018 Apr 2]. Available from: http://blumcenter.berkeley.edu/news-posts/fighting-for-the-last-mile-of-womens-rights-the-head-of-un-women-and-uc-berkeley-gender-scholars-look-toward-2015-2030/

[27] Escobar-Chaves SL, Tortolero SR, Mâsse LC, et al. Recruiting and retaining minority women: findings from the women on the move study. Ethn Dis. 2002 Spring;12:242–251. 12019934

[28] Wendler D, Kington R, Madans J, et al. Are racial and ethnic minorities less willing to participate in health research? PLoS Med. 2006;3:e19. 16318411 10.1371/journal.pmed.0030019 PMC1298944

[29] Miret C, Gonzalez C, Prats-Uribe A, et al. Factors that influence compliance with hand hygiene in healthcare professionals: a concept mapping study. J Healthcare Qual Res. 2020;35:103–112. 10.1016/j.jhqr.2019.09.003 32179017

[30] Wee S, Todd MJ, Oshiro M, et al. Modifiers of neighbors’ bystander intervention in intimate partner violence: a concept mapping study. Violence Gender. 2016;3:55–63. 27626038 10.1089/vio.2015.0012 PMC4997712

[31] Kim MS. Concept mapping of career motivation of women with higher education. Front Psychol. 2020;11:1073. 32536894 10.3389/fpsyg.2020.01073 PMC7269102

[32] Howell BM, Seater M, McLinden D. Using concept mapping methods to define “healthy aging” in Anchorage, Alaska. J Appl Gerontol. 2020 Jan 8:733464819898643. DOI:10.1177/0733464819898643. 31910705

[33] Pintoi AJ, Zeitz HJ. Concept mapping: a strategy for promoting meaningful learning in medical education. Med Teach. 1997;19:114–121.

[34] Felx A, Kane M, Corbiere M, et al. Using group concept mapping to develop a conceptual model of housing and community-based residential settings for adults with severe mental illness. Front Psychiatry. 2020;11:430. 32636764 10.3389/fpsyt.2020.00430 PMC7319103

[35] Dunlop S, Lewis N, Richardson R, et al. Using group concept mapping to explore the complexities of managing children’s care. Nurse Res. 2020 Jan 16. Epub ahead of print. DOI:10.7748/nr.2020.e1696. 31942785

[36] Sommerfeld DH, Jaramillo ET, Lujan E, et al. Health care access and utilization for American Indian elders: a concept-mapping study. J Gerontol B Psychol Sci Soc Sci. 2019;76:141–151. 10.1093/geronb/gbz112 PMC7756708 31587056

[37] Fornells A, Rodrigo Z, Rovira X, et al. Promoting consensus in the concept mapping methodology: an application in the hospitality sector. Pattern Recognit Lett. 2015;67:39–48.

[38] Daley BJ, Durning SJ, Torre DM. Using concept maps to create meaningful learning in medical education. MedEdPublish. 2016;5:19.

[39] Yaphe H, Adekoya I, Steiner L, et al. Exploring the experiences of people in Ontario, Canada who have trouble affording medicines: a qualitative concept mapping study. BMJ Open. 2019;9:e033933. 10.1136/bmjopen-2019-033933 PMC6937130 31888944

[40] Kane M, Rosas S. Conversations about group concept mapping: applications, examples and enhancements. California: Sage Publications Inc; 2018. Chapter 3, GCM in action - analyzing the results; p. 41–70.

[41] Kruskall J, Wish M. Multidimensional scaling. USA: Sage Publications Inc; 1978.

[42] Davison ML. Introduction to multidimensional scaling and its applications. Appl Psychol Meas. 1983;7:373–379.

[43] Anderberg MR. Cluster analysis for applications. 1st ed. Probability and Mathematical Statistics: a series of monographs and textbooks. USA: Academic Press; 1973.

[44] Everitt B. Cluster analysis. 2nd ed. New York: Halsted Press; 1980.

[45] Hair JF, Tatham RL, Anderson RE, et al. Multivariate data analysis. 5th ed. USA: Prentice Hall Publishers; 1998.

[46] Health Equity. Toronto: Public Health Ontario; 2019 [cited 2020 Aug 29]. Available from: https://www.publichealthontario.ca/en/health-topics/health-equity

[47] United Nations. Universal declaration on human rights. Geneva: United Nations; 1948 [cited 2020 Aug 29]. Available from: https://www.un.org/en/universal-declaration-human-rights/

[48] United Nations. United Nations declaration on the rights of indigenous peoples. Geneva: United Nations; 2007 [cited 2020 Aug 27]. Available from: https://www.un.org/development/desa/indigenouspeoples/declaration-on-the-rights-of-indigenous-peoples.html

[49] Office of The High Commissioner on Human Rights. UN convention on the rights of the child. Geneva: United Nations; 1990 [cited 2020 Aug 27]. Available from: https://www.ohchr.org/en/professionalinterest/pages/crc.aspx

[50] United Nations Department of Economic and Social Affairs. Convention on the rights of persons with disabilities. Geneva: United Nations Department of Economic and Social Affairs; 2006 [cited 2020 Aug 27]. Available from: https://www.un.org/development/desa/disabilities/convention-on-the-rights-of-persons-with-disabilities.html

[51] Braveman P, Gottlieb L. The social determinants of health: it’s time to consider the causes of the causes. Public Health Rep. 2014;129:19–31. 10.1177/00333549141291S206 PMC3863696 24385661

[52] Kothari A, Wathen CN. A critical look at integrated knowledge translation. Health Policy. 2013;109:187–191. 23228520 10.1016/j.healthpol.2012.11.004

[53] Chapman S. Advocacy in public health: roles and challenges. Int J Epidemiol. 2001;30:1226–1232. 11821312 10.1093/ije/30.6.1226

[54] Frenk J. The global health system: strengthening national health systems as the next step for global progress. PLoS Med. 2010;7:e1000089. 20069038 10.1371/journal.pmed.1000089 PMC2797599

[55] WHO [Internet]. Health systems. Geneva: WHO; 2020 [cited 2020 Aug 30]. Available from: https://www.who.int/healthsystems/about/en/

[56] WHO. Everybody business: strengthening health systems to improve health outcomes: WHO’s framework for action. Geneva: WHO; 2006 [cited 2020 Aug 30]. Available from: https://www.who.int/healthsystems/strategy/everybodys_business.pdf?ua=1

[57] Jost CC, Mariner JC, Roeder PL, et al. Participatory epidemiology in disease surveillance and research. Rev Sci Tech. 2007;26:537–547. 18293603

[58] Neal P, Knowles A, DuMond S. Fostering community engagement and acquiring understanding of health needs through a participatory rural appraisal in Haiti. Prog Community Health Partners. 2018;12:389–394. 10.1353/cpr.2018.0064 30739893

[59] Ospina-Pinillos L, Davenport T, Mendoza Diaz A, et al. Using participatory design methodologies to co-design and culturally adapt the Spanish version of the mental health eClinic: qualitative study. J Med Internet Res. 2019 Aug 2;21:e14127. 31376271 10.2196/14127 PMC6696860

[60] Seino S, Kitamura A, Tomine Y, et al. A community-wide intervention trial for preventing and reducing frailty among older adults living in metropolitan areas: design and baseline survey for a study integrating participatory action research with a cluster trial. J Epidemiol. 2019;29:73–81. 29962492 10.2188/jea.JE20170109 PMC6336723

